# Long Non-Coding RNAs As Potential Novel Prognostic Biomarkers in Colorectal Cancer

**DOI:** 10.3389/fgene.2016.00054

**Published:** 2016-04-12

**Authors:** Ester Saus, Anna Brunet-Vega, Susana Iraola-Guzmán, Cinta Pegueroles, Toni Gabaldón, Carles Pericay

**Affiliations:** ^1^Centre for Genomic Regulation, The Barcelona Institute of Science and TechnologyBarcelona, Spain; ^2^Universitat Pompeu FabraBarcelona, Spain; ^3^Department of Oncology Research, Parc Taulí Foundation, Corporació Sanitària Parc Taulí - University Institute - UAB BarcelonaSabadell, Spain; ^4^Institució Catalana de Recerca i Estudis AvançatsBarcelona, Spain; ^5^Oncology Service, Hospital de Sabadell, Corporació Sanitària Parc Taulí - University Institute - UAB BarcelonaSabadell, Spain

**Keywords:** long non-coding RNA, ncRNA, prognostic marker, colorectal cancer, biomarker

## Abstract

Colorectal cancer (CRC) is the fourth most common cause of death worldwide. Surgery is usually the first line of treatment for patients with CRC but many tumors with similar histopathological features show significantly different clinical outcomes. The discovery of robust prognostic biomarkers in patients with CRC is imperative to achieve more effective treatment strategies and improve patient's care. Recent progress in next generation sequencing methods and transcriptome analysis has revealed that a much larger part of the genome is transcribed into RNA than previously assumed. Collectively referred to as non-coding RNAs (ncRNAs), some of these RNA molecules such as microRNAs (miRNAs) and long non-coding RNAs (lncRNAs) have been shown to be altered and to play critical roles in tumor biology. This discovery leads to exciting possibilities for personalized cancer diagnosis, and therapy. Many lncRNAs are tissue and cancer-type specific and have already revealed to be useful as prognostic markers. In this review, we focus on recent findings concerning aberrant expression of lncRNAs in CRC tumors and emphasize their prognostic potential in CRC. Further studies focused on the mechanisms of action of lncRNAs will contribute to the development of novel biomarkers for diagnosis and disease progression.

## Introduction

### Need for prognostic markers in colorectal cancer

Colorectal cancer (CRC) is among the most commonly diagnosed cancers worldwide. In 2012, according to the GLOBOCAN estimations, there were 1.3 million new cases of CRC worldwide (Ferlay et al., 2015). That year, around 694,000 patients died of the disease, accounting for 8.5% of all cancer deaths, and ranking CRC the fourth most common cause of death from cancer. Colorectal cancer develops as a result of the pathologic transformation of normal colonic epithelium to adenomatous polyp, which ultimately leads to invasive cancer (Jass, 2006). Tumor induction and progression is considered a stepwise process with the accumulation of different genetic and epigenetic alterations. Most tumors (85%) are generated by chromosomal instability and are associated with high frequency aneuploidy and allelic imbalance. The prognosis and survival of CRC patients are clearly related to the degree of penetration of the tumor through the bowel wall, and to the presence or absence of nodal involvement and distant metastases. The American Joint Committee on Cancer's TNM (Tumor size, lymph Node involvement, and distant Metastasis) staging system is the current standard for determining the prognosis of patients with CRC. After diagnosis, staging (stages I to IV) is performed based on the extent of penetration of the colorectal cancer into the layers forming the wall of the colon and whether the tumor cells have spread to lymph nodes and other organs. This classification is important for planning patient treatment. The gold standard therapy for resectable tumors is surgery, whereas adjuvant chemotherapy is indicated only in patients with a high risk of disease recurrence. Traditional prognostic factors for malignancy include clinical and pathological criteria such as patient's age, tumor size, and tumor grade, number of affected local lymph nodes, and presence and grade of metastasis. Although these factors have been used for decades, they have serious limitations in predicting patient outcome (Burke, 2004). In stage II the tumor is confined to the bowel wall in contrast to stage III and IV, in which the tumors have disseminated to local lymph nodes and distant organs, respectively. The majority of patients with stage II do not receive adjuvant chemotherapy treatment since, at present there is no evidence for beneficial effect for this patient group as a whole. From all those patients receiving treatment, only a small proportion (1–5%) derives clinical benefit, whereas adverse side effects are common (Aranha and Benson, 2007). Nevertheless, 20–25% of all stage II CRC patients will present with recurrent disease and subsequent death from disease within 5 years after primary surgery (Benson et al., 2004). On the other hand, adjuvant treatment is universally recommended for all patients with stage III disease, for whom the cancer has already spread to the surrounding lymph nodes.

Thus, there is a need to add prognostic and predictive markers to the current staging system. In the last two to three decades, hundreds of cancer biological prognostic markers for CRC cancer have been proposed such as, microsatellite instability (Popat et al., 2005), chromosomal instability (Pino and Chung, 2010), K-ras mutation (Rui et al., 2013), mRNA and microRNA expression profile (Nugent et al., 2011; Salazar et al., 2011; Sanz-Pamplona et al., 2012). Although they have shown a potential in this field, validation studies are still required and, to date, there is insufficient evidence to recommend the routine clinical use of any of these putative biomarkers. Therefore, the discovery of robust prognostic and/or predictive biomarkers in patients with CRC is imperative for advancing treatment strategies for the disease and improving patient care.

In this review, we provide an overview of recent findings concerning the prognostic potential of lncRNAs in CRC.

### LncRNAs in cancer and their potential as prognostic biomarkers

Recent progress in next generation sequencing methods and in transcriptome analysis has shown that a much larger portion of the genome is transcribed into RNA than previously assumed (Consortium, 2012). Long non-coding RNAs (lncRNAs) are a widespread class of expressed RNA transcripts longer than 200 nucleotides, that have no coding potential but that, similar to protein coding genes, are typically polyadenylated, transcribed by RNA polymerase II, and spliced (Ponting et al., 2009; Ulitsky and Bartel, 2013). Compared to protein-coding genes, they are expressed at lower levels and in a more tissue-specific manner (Derrien et al., 2012). Initially, lncRNAs were thought to be simply the result of transcriptional noise, but recent studies point to a crucial involvement of a growing number of lncRNAs in central cellular processes such as epigenetic modulation, transcription, and translation (Mercer and Mattick, 2013; Johnsson et al., 2014). During the last decade, the rapid advance in sequencing and array technologies has further facilitated the study of these molecules regarding their involvement in pathological processes. In this context, recent studies show a clear implication of some lncRNAs in the development of various diseases, ranging from neurodegeneration to cancer (Wapinski and Chang, 2011).

Recent large-scale sequencing efforts such as the International Cancer Genomics Consortium and the Cancer Genome Atlas, as well as numerous small-scale projects, have focused on the detection of somatic genetic and epigenetic alterations that are tightly associated with the disease. These studies have revealed that a significant fraction of such alterations lie in non-coding parts of the genome, often affecting lncRNA loci. Such alterations can involve deletions, differences in copy number, point mutations but also regulatory changes that can lead through different modes of action to changes in gene expression. For example, *MALAT1*, a lncRNA with an important role in cell proliferation, migration and invasion, has been found significantly up-regulated in several cancers, including lung, breast, prostate, liver, and colon. Many cancer-relevant genes, in particular tumor suppressor genes, encompass long antisense ncRNAs (Nie et al., 2012), and some evolutionarily conserved non-coding transcripts are altered in human cancer, such as leukemia and other carcinomas (Calin et al., 2007). The finding that alterations in sequence or expression of some lncRNAs might be associated to specific stages of cancer progression paves the way for their use as prognostic biomarkers (Hu et al., 2014).

Several studies have indicated dysregulation of lncRNA expression in CRC tumors and a potential functional impact in pathogenesis and clinical implications. These studies are quite heterogeneous, including association studies in large-scale sequencing projects as well as detailed analyses of the direct involvement of particular lncRNAs in the progression of the disease. Overall, the precise molecular mechanisms underlying CRC pathogenesis remain to be fully elucidated, with still a limited number of lncRNAs with a well-characterized molecular mechanism of action. The review of these few known molecular mechanisms is beyond the scope of this paper, and we direct the interested reader to recent reviews in that topic (Gutschner and Diederichs, 2012; Zhang K. et al., 2014). Besides, there are a growing number of new lncRNAs with unknown functions, and whose implications in tumor biology are not fully understood. For instance, a recent study based on 7256 RNA-seq libraries from tumors, normal tissues and cell lines, identified a total of 196 non-coding transcripts associated with CRC, but the vast majority of them (163) were previously unknown (Iyer et al., 2015). Imbalances in expression levels may be used to identify new potential markers for disease progression. However, further analyses will be needed to address whether alterations in sequence or expression of some lncRNAs are involved in special biological processes relevant to cancer.

**Figure 1:**
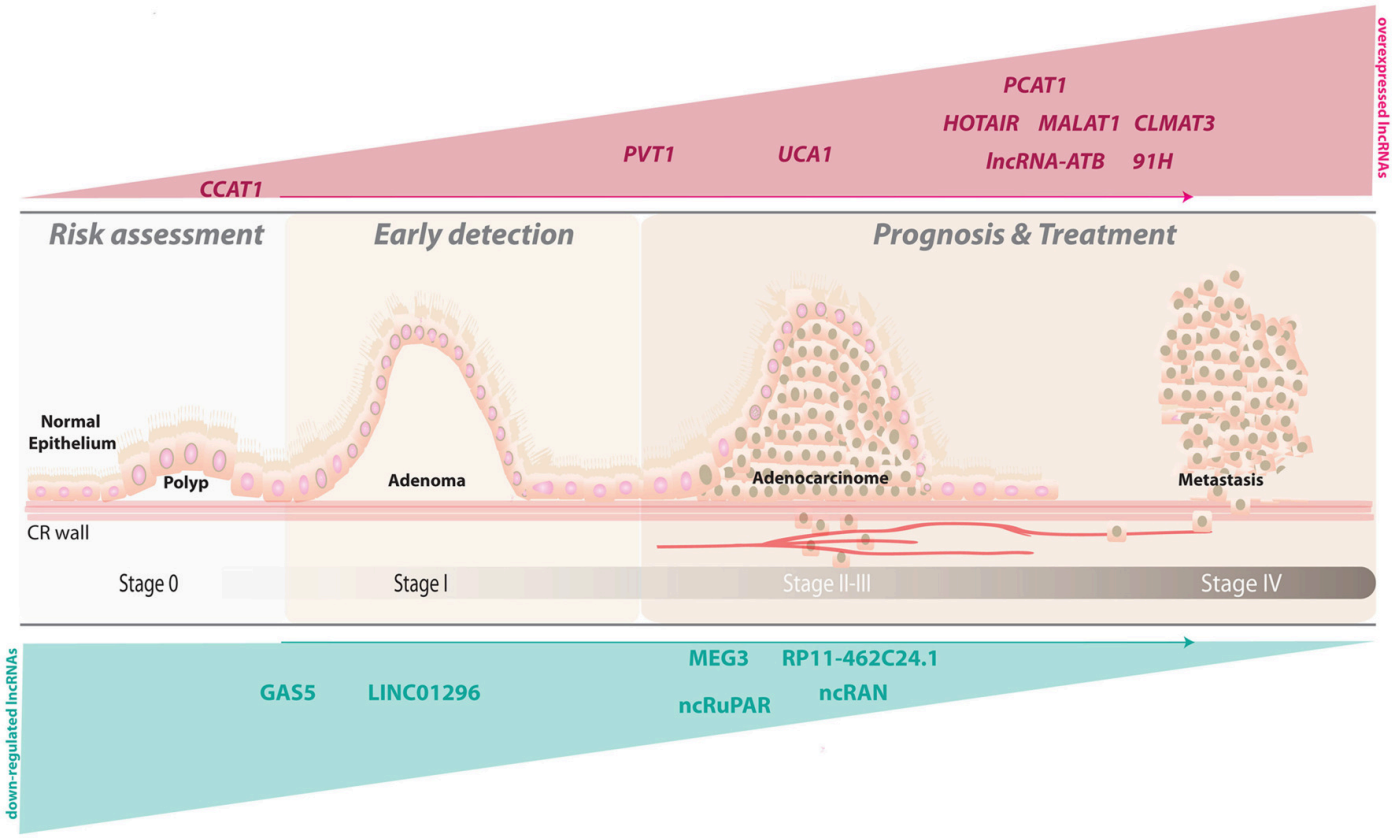
**LncRNAs as potential prognostic biomarkers for human colorectal cancer**. A depiction of a sagittal view of the colorectal wall showing the progression of lesions associated with CRC and the accompanying alterations in the observed expression levels for several lncRNAs.

In the present review, we focus on studies investigating whether lncRNAs can serve as prognostic biomarkers for CRC (Table 1; Figure 1), and we will discuss recent advances in the potential value of lncRNAs to predict outcomes of CRC patients (Table 2). The candidate lncRNAs reviewed below may add prognostic power to the current gold-standard AJCC (American Joint Committee on Cancer) staging system.

**Table 1:** **Description of potential prognostic lncRNA markers in colorectal cancer**.

**LncRNA**	**Genomic location**	**Gene size (kb)**	**Locus**	**Type**	**Function in tumorigenesis of CRC and mechanism of action**	**References**
**ONCOGENIC lncRNA**						
***91H***	Chr11p15	119.32	*H19/IGF2*	AS	Proliferation, migration and invasion.	Deng et al., 2014
***CCAT1***	Chr8q24.21	11.88	*c-MYC*	–	Progression and metastasis mediated by c-Myc binding to *CCAT1* promoter region.	Nissan et al., 2012; Alaiyan et al., 2013; He et al., 2014; Zhao et al., 2015
***CLMAT3***	Chr14q32.31	1.55	*SPARC*	–	Progression and metastasis.	Ye et al., 2015
***DANCR***	Chr4q12	7.94	–	–	Progression and metastasis.	Liu et al., 2015
***FEZF1-AS1***	Chr7q31.32	6.42	*FEZF1*	AS	Proliferation, migration and invasion through S-phase entry.	Chen et al., 2016
***FTX***	ChrXq13.2	329.62	*XIC*	lincRNA	Proliferation, migration, and invasion mediated by *Xist* up-regulation.	Guo X. B. et al., 2015
***HOTAIR***	Chr12q13.13	12.64	*HOXC*	AS	Migration, and invasion through Polycomb-mediated chromatin modifications, and EMT molecules interaction.	Rinn et al., 2007; Kogo et al., 2011; Wu Z. H. et al., 2014; Zhang S. et al., 2014; Zhao et al., 2015
***HOTTIP***	Chr7p15.2	8.68	*HoxA*	AS	Progression and metastasis through G1 phase arrest, S phase reduction, and apoptosis promotion through p21 inactivation.	Ren et al., 2015; Lian et al., 2015
***lncRNA-ATB***	Chr14q11.2	2.73	–	–	Promotion of invasion and metastasis via interaction with transcription factors ZEB1 and ZEB2, involved in EMT in other cancer types.	Yuan et al., 2014; Iguchi et al., 2015
***MALAT1***	Chr11q13.1	8.75	*NEAT-2*	lincRNA	Proliferation, migration, and invasion through release of proto-oncogene *PTBP2*, and interaction with AKAP-9 protein, and Wnt/β-catenin signaling pathway proteins.	Ji et al., 2013; Smolle et al., 2014; Ji et al., 2014; Yang et al., 2015
***PCAT1***	Chr8q24.21	173.96	–	lincRNA	Potential promotion of metastasis through interaction with *BRCA2* and PRC2 in other cancer types.	Prensner et al., 2011; Ge et al., 2013
***PVT1***	Chr8q24.21	306.72	*PVT1*	lincRNA	Proliferation, invasion, and antiapoptotic effect via modulation of chromatin remodeling complex SWI/SNF.	Takahashi et al., 2014
***TUG1***	Chr22q12.2	9.7	*TUG1*	lincRNA	Progression and metastasis mediated by modulation of the expression of EMT related genes.	Sun et al., 2016
***UCA1***	Chr19p13.12	7.37	*UCA1*	lincRNA	Proliferation, and antiapoptotic effect through G0/G1 growth arrest.	Han et al., 2014
**TUMOR SUPPRESSIVE lncRNA**						
***GAS5***	Chr1q25.1	4.98	*GAS5*	–	Inhibition of cell proliferation by mimicking glucocorticoid response element (GRE).	Mourtada-Maarabouni et al., 2010; Yin et al., 2014
***LINC01296***	Chr22q11.1	20.55	–	lincRNA	Potential epigenetic silencing mechanism.	Qiu and Yan, 2015
***MEG3***	Chr14q32.2	81.62	*DLK1-MEG3*	lincRNA	Prevents abnormal proliferation by promoting p53 expression.	Zhang et al., 2003; Zhou et al., 2007; Yin et al., 2015
***NcRAN***	Chr17q25.1	7.58	*SNHG16*	AS	Involved in migration and invasion.	Qi et al., 2015; Smolle et al., 2014
***ncRuPAR***	Chr5q13.3	0.48	*ncRuPAR*	lncRNA	Involved in invasion and metastasis.	Yan et al., 2014
***RP11-462C24.1***	Chr4q25	82.27	*RPL34*	AS	Involved in invasion, and metastasis.	Shi et al., 2014
***TUSC7***	Chr3q13.31	14.34	*LSAMP*	lincRNA	Growth suppressor through regulation of apoptotic and cell cycle transcripts in osteoblasts. No mechanism described in CRC.	Qi et al., 2013

Abbreviations: AS, antisense; EMT, Epithelial-mesenchymal transition; lincRNA, long intergenic non-coding RNA.

**Table 2:** **Compilation of studies showing significant associations between the expression of lncRNAs with CRC clinicopathological features**.

**LncRNA**	**Study Design**	**Sample size (cohort/validation)**	**Detection method**	**lncRNA expression in CRC**	**Clinicopathological features associated to CRC**	**Overall survival rate**	**References**
**ONCOGENIC lncRNA**							
***91H***	Cohort	72	qRT-PCR^1^	up-regulated	Poor prognosis, DM	HR = 3.66, *P* = 0.001	Deng et al., 2014
***CCAT1***	Cohort	113	qRT-PCR, ISH	up-regulated	Progression, LNM, LM	ND	Alaiyan et al., 2013
***CCAT1***	Cohort	48	qRT-PCR^1^	up-regulated	TNM stage, LNM, poor OS	Kaplan-Meier *P* < 0.001	He et al., 2014
***CLMAT3***	Cohort	30/90	qRT-PCR^1^	up-regulated	LM, poor OS	HR = 2.05, *P* = 0.02	Ye et al., 2015
***DANCR***	Cohort	104	qRT-PCR^1^	up-regulated	TNM stage, LNM	HR = 2.131, *P* = 0.009	Liu et al., 2015
***FEZF1-AS1***	Cohort	34 pairs / 153	qRT-PCR, ISH	up-regulated	TNM stage, LNM, DM	HR = 2.401, *P* = 0.035	Chen et al., 2016
***FTX***	Cohort	35 pairs / 187	qRT-PCR	up-regulated	TNM stage, LI, VI	HR = 2.37, *P* = 0.00041	Guo X. B. et al., 2015
***HOTAIR***	Cohort	100 /320	qRT-PCR^2^, EA	up-regulated	LM, poor prognosis	HR = 5.62, *P* = 0.008	Kogo et al., 2011
***HOTAIR***	Cohort	152	qRT-PCR^1^	up-regulated	LNM	HR = 3.915, *P* = 0.021	Wu Z. H. et al., 2014
***HOTAIR***	Meta-analysis	2,033	qRT-PCR, EM	up-regulated	Poor prognosis	^*^HR = 2.26 (95%CI: 1.62–3.15)	Zhang S. et al., 2014
***HOTTIP***	Cohort	156 / 21	qRT-PCR^1^	up-regulated	TNM stage, DM	HR = 2.151, *P* = 0.017	Ren et al., 2015
***lncRNA-ATB***	Cohort	124	qRT-PCR^2^	up-regulated	TS, IC, LI, VI, LNM	Kaplan-Meier *P* = 0.036	Iguchi et al., 2015
***MALAT1***	Cohort	147	qRT-PCR^2^	up-regulated	Poor prognosis	HR = 3.968, *P* = 0.002	Zheng et al., 2014
***MALAT1***	Meta-analysis	1033	qRT-PCR, ISH	up-regulated	Poor OS, LNM, DM	HR = 2.05, *P* = 0.01	Wu et al., 2015
***PCAT-1***	Cohort	108	qRT-PCR^1^	up-regulated	Poor prognosis and DM	HR = 3.12, *P* = 0.007	Ge et al., 2013
***PVT-1***	Cohort	164	qRT-PCR^2^	up-regulated	Poor prognosis	HR = 2.532, *P* = 0.016	Takahashi et al., 2014
***TUG1***	Cohort	120	qRT-PCR	up-regulated	-	Kaplan-Meier *P* < 0.001	Sun et al., 2016
***UCA1***	Cohort	80	qRT-PCR^1^	up-regulated	Poor prognosis	Kaplan-Meier *P* = 0.046	Han et al., 2014
***UCA1***	Cohort	54	qRT-PCR^1^	up-regulated	Poor prognosis, LM, DM, TNM	HR = 3.13, *P* = 0.023	Ni et al., 2015
**TUMOR SUPPRESSIVE lncRNA**							
***GAS5***	Cohort	66	qRT-PCR^1^	down-regulation	Poor OS	HR = 0.036, *P* = 0.034	Yin et al., 2014
***LINC01296***	Meta-analysis	160/541	EA	down-regulation	–	HR = 0.43, *P* = 0.001	Qiu and Yan, 2015
***MEG3***	Cohort	62	qRT-PCR^1^	down-regulation	TNM stage, tumor invasion	HR = 0.133, *P* = 0.049	Yin et al., 2015
ncRAN^**^	Cohort	80	qRT-PCR^1^	down-regulation	–	^*^HR = 3.872, *P* = 0.024	Qi et al., 2015
***ncRuPAR***	Cohort	105	qRT-PCR	down-regulation	–	ND	Yan et al., 2014
***RP11-462C24.1***	Cohort	86	qRT-PCR^1^	down-regulation	Poor prognosis and DM	HR = 0.056, *P* < 0.005	Shi et al., 2014
***TUSC7***	Cohort	81	qRT-PCR^1^	down-regulation	TS, TNM stage, DM	HR = 3.201, *P* = 0.019	Qi et al., 2013

*Abbreviations: ISH, In-situ hybridization; EM, expression microarray; EA, Exon array; qRT-PCR, quantitative real-time PCR using SYBR Green (1) or probe-based detection (2); TNM, tumor size, lymph node involvement, and distant metastasis classification; DM, distant metastasis; LNM, lymph node metastasis; OS, overall survival; LM, liver metastasis; TS, tumor size; IC, colorectal wall invasion; LI, lymphatic invasion; VI, vascular invasion; HR, hazardous risk; P, p-value; 95%CI, 95% confidence interval; ND, not determined*.

* *Pooled data from a meta-analysis*.

** *The analysis was carried out considering only ncRAN transcript Nbla10727*.

## Oncogenic LncRNAs associated with prognosis in colorectal cancer

### 91H

The “H19 antisense long, non-coding RNA” *91H* is a 120 kb antisense long non-coding transcript spanning the *H19* gene locus. *91H* expression has been associated with breast cancer (Berteaux et al., 2008) and esophageal squamous cell carcinoma progression (Gao et al., 2015). Deng et al. (2014) found *91H* transcript over-expression in CRC tissues compared with adjacent non-cancerous tissue, as well as CRC cell lines compared with normal intestinal epithelial cell line. The authors showed that *in vitro* knockdown of *91H* inhibited cell motility, migration and invasiveness of CRC cells lines. Finally, they observed that elevated expression of *91H* was associated with poor prognosis and distant metastasis in patients with CRC (Deng et al., 2014).

### CCAT1

Colon Cancer Associated Transcript-1 (*CCAT1*) was first described as an 11.88 kb lncRNA highly expressed in CRC compared to normal tissue (Nissan et al., 2012). *CCAT1* is transcribed from a locus in chromosome 8q24.21, located 515 kb upstream of the well-known transcription factor *c-Myc*, and reported to be a “hot-spot” locus with many genetic alterations in colorectal cancer (Zanke et al., 2007; Haerian et al., 2011). Recently, Qiu and Yan (2015) analyzed a cohort of five published datasets including 355 samples, validating the association of *CCAT1* with CRC (Qiu and Yan, 2015). *CCAT1* up-regulation is present in the vast majority of primary CRC tumors, including pre-cancerous polyps (adenomas), lymph nodes, blood, and distant CRC metastasis. Increased expression of *CCAT1* has been correlated with patient's clinical stage, lymph node metastasis, and survival time. Taking the information together, it suggests that *CCAT1* could have key roles in both tumorigenesis and metastasis, and thus it may be a good candidate as a clinical outcome biomarker (Alaiyan et al., 2013; He et al., 2014). Indeed, a recent study showed that *CCAT1* levels were not only significantly higher in plasma of CRC patients compared to control individuals but also significantly decreased in CRC patients after surgical treatment (Zhao et al., 2015). The authors established by means of ROC analysis that plasma *CCAT1* provided a strong separation between CRC patients and the control group (AUC = 0.836, *P* < 0.001), an effective screening which improved when combining plasma levels of *CCAT1* and *HOTAIR* (AUC = 0.954, *P* < 0.001). In addition, this combination of *CCAT1* and *HOTAIR* levels in plasma could detect CRC at an early stage (85%), demonstrating that increased plasma levels of *CCAT1* (especially when combined with *HOTAIR*) could be used as a predictive biomarker for CRC screening (Zhao et al., 2015).

### CLMAT3

Cancer Liver Metastasis Associated Transcript-3 (*CLMAT3*) is a long non-coding RNA that recently emerged as a potential independent prognostic factor in CRC with liver metastasis (Ye et al., 2015). Almost half of the CRC patients with advanced CRC will suffer colorectal liver metastasis during the disease course, and so far there are no available early prognostic biomarkers that enable the screening of CRC patients suffering liver metastasis. To that end, a new study aimed to profile the expression levels of lncRNAs in a total of 264 CRC samples with or without liver metastasis (synchronous and metachronous). First, the authors identified 40 lncRNAs differentially expressed in the discovery phase. Next, they validated three novel lncRNAs (*TR140014124, TR01015341*, and *TR05005298*, further termed *CLMAT1* to *3* respectively) by RT-qPCR in an independent cohort. Finally, they found that lncRNA *CLMAT3* located in chromosome 14 (chr14:101379770-101381326, Hg19) was the most up-regulated lncRNA and that its high expression was significantly associated with liver metastasis and lymph node metastasis. In addition, the patients with tumors displaying high lncRNA-*CLMAT3* expression exhibited shorter overall survival than those with low lncRNA-*CLMAT3* expression tumors. Furthermore, the results of multivariate analysis indicated that lncRNA-*CLMAT3* expression was an independent prognostic indicator of CRC patient's survival compared with CRC patients with liver metastasis (Ye et al., 2015). However, these results need to be proven in a larger cohort, including a heterogeneous population, and whose 5-year overall survival rate is confirmed.

### DANCR

Differentiation Antagonizing Non-Protein Coding RNA (*DANCR*) is a 7.94 RNA gene, mapping on chr4q12, and associated to stem cell-like state. It has been shown to be involved in maintenance of undifferentiated cell state of epidermis (Kretz et al., 2012) and osteoblasts (Zhu and Xu, 2013; Jia et al., 2015). Two recent studies suggest a novel mechanism of tumorigenesis involving this lncRNA in hepatocellular carcinoma (Yuan et al., 2016) and in colorectal cancer (Liu et al., 2015), respectively. The former shows that *DANCR* action may increase stemness features and offer a potential prognostic marker and a therapeutic target for hepatocellular carcinoma. The latter quantifies the expression of lncRNA *DANCR* in a total of 104 CRC tissues and adjacent non-tumor tissues by qRT-PCR, and analyzes the correlation with the clinicopathological characteristics and outcome of the CRC patients included in the study. As a result, the authors show a remarkably higher expression of lncRNA *DANCR* in CRC tissues compared with adjacent non-tumor tissues (*P* < 0.05), and show that these values are significantly associated with TNM stage, histologic grade and lymph node metastasis (*P* < 0.05), with no significant correlation with other clinical features such as gender, age, tumor size, and local invasion (*P*>0.05). Moreover, the authors suggest that lncRNA *DANCR* expression could be an independent prognostic indicator for overall survival (HR = 2.131, 95% CI, 1.157–7.058; *P* = 0.009) and disease-free survival (HR = 2.397, 95% CI, 1.385–7.279; *P* = 0.006) in patients with colorectal cancer. Thus, lncRNA *DANCR* up-regulation is associated with aggressive progression and poor prognosis in colorectal cancer and may function as an independent marker for predicting the clinical outcome of colorectal cancer patients. However, further studies are needed to elucidate the mechanisms of action of lncRNA *DANCR* in colorectal cancer.

### FEZF1-AS1

FEZ family zinc finger 1 antisense RNA 1 (*FEZF1-AS1*) is a recently identified lncRNA which is located in chr7q31.32 on the opposite strand of gene *FEZF1*. Although its function in normal or cancer cells is still unknown, Chen et al. (2016) have recently identified significantly higher *FEZF1-AS1* expression levels in tumor tissues of CRC patients compared to adjacent non-neoplastic mucosa tissues (*P* = 0.004). Specifically, in patients diagnosed with lymph node metastases, the relative mean expression of *FEZF1-AS1* was over 2.30 fold higher than in patients without metastases, indicating that *FEZF1-AS1* might have a fundamental role in CRC metastasis. Moreover, the authors reported that the up-regulation of lncRNA *FEZF1-AS1* was significantly associated with aggressive phenotypes of CRC and poor prognosis in patients with CRC when using Kaplan-Meier analysis. High expression levels of *FEZF1-AS1* in CRC were significantly correlated with overall survival (*P* = 0.002) and disease-free survival (*P* = 0.002) of CRC patients, with high levels of *FEZF1-AS1* being associated with short survival times. Further univariate and multivariate analyses indicated that high-level expression of *FEZF1-AS1* was an independent prognostic factor of outcomes in patients with CRC (HR = 2.401, *P* = 0.035). In a further step, the authors showed that *FEZF1-AS1* increases cell proliferation, migration and invasiveness by means of *in vitro* studies in a panel of CRC cell lines. In addition, *FEZF1-AS1* down-regulation *in vivo* inhibited tumor growth and metastasis, pointing to a promotional role of *FEZF1-AS1* in CRC tumorigenesis and progression. Thus, all the results together suggest lncRNA *FEZF1-AS1* as a putative prognostic biomarker and a target for new therapies of CRC (Chen et al., 2016).

### FTX

The *FTX* transcript is a conserved functional lncRNA encoded within the X-inactivation center (XIC). This gene located in chrXq13.2 harbors 4 miRNAs in its introns, the miR-374b/421 cluster and the miR-545/374a cluster. Guo X. B. et al. (2015) measured lncRNA *FTX* expression in more than 100 CRC samples and found that lncRNA *FTX* was significantly up-regulated in CRC tissues compared with adjacent normal tissues. High lncRNA *FTX* expression correlated with differentiation grade, lymph vascular invasion and clinical stage. However, there were no significant correlations with other clinicopathological features, such as age, sex, tumor histology, perineural invasion, tumor site, and lymph node metastasis. Multivariate regression analysis indicated that the status of lncRNA *FTX* expression was an independent predictor of overall survival, as well as differentiation grade and clinical stage, indicating that lncRNA *FTX* may be a promising prognostic biomarker for CRC patients.

### HOTAIR

HOX Transcript Antisense Intergenic RNA *(HOTAIR)* is a 2.2 kb long non-coding RNA transcribed from the antisense strand of *HOXC* gene cluster, which is located in chromosome 12q13, and which takes part in the epigenetic regulation of gene expression (Gupta et al., 2010). Several studies have shown that *HOTAIR* is dysregulated in many cancers, including colorectal carcinomas (Gutschner and Diederichs, 2012; Wu Y. et al., 2014; Hajjari and Salavaty, 2015). Kogo et al. (2011) measured *HOTAIR* expression in 100 colon cancer tissue samples along with their matched normal tissues and reported significant differences between them. In addition, they detected a strong association between high *HOTAIR* expression and liver metastasis and poor patient prognosis. Moreover, cDNA microarrays from a specific subgroup of CRC samples obtained by laser micro-dissection suggested that *HOTAIR* expression induced genome-wide re-targeting of the regulator Polycomb Repressive Complex 2 (*PRC2*). Furthermore, *in vitro* studies confirmed invasion promotion by *HOTAIR* over-expression (Kogo et al., 2011). All these studies suggest that changes in *HOTAIR* expression may impact the expression of multiple genes by the loss of cooperation with *PRC2* complex, resulting in an increase of undifferentiated cancer cells. Thus, *HOTAIR* is a valuable factor for colorectal cancer prognosis. Moreover, *HOTAIR* can promote colorectal cancer cell migration and invasiveness and may participate in epithelial-mesenchymal transition. Further studies are warranted to advance our understanding of the involvement of *HOTAIR* in cancer development, since this lncRNA is a potential target for cancer prevention and treatment (Wu Z. H. et al., 2014). A total of 19 papers comprising 2033 patients were included into this meta-analysis. The authors found that *HOTAIR* expression was associated with a poorer prognosis in patients with different types of cancer, as well as advanced pathological stage (Zhang S. et al., 2014). Finally, Zhao et al. (2015) have recently found levels of *HOTAIR* significantly higher in plasma of CRC patients than that of the healthy controls. In the same study, the authors also reported that plasma levels of *HOTAIR* were significantly decreased in CRC patients after surgical treatment as compared to pre-operative patients, confirming, all together, the potential use of *HOTAIR* as a diagnostic biomarker for CRC.

### HOTTIP

*HOXA* transcript at the distal tip (*HOTTIP*) is a ~3.8 kb lncRNA, which is spliced and polyadenylated. This gene is located at 5′ end of *HOXA* cluster and regulates the transcription of *HOXA* genes *in vivo*, which are collinearly expressed along the antero-posterior axis (Wang et al., 2011). The coordinated expression of *HOTTIP* and the adjacent *HOXA* genes has been observed in normal cells, tumors and also cancer cell lines (Sasaki et al., 2007). *HOTTIP* is expressed at low levels (~0.3 copies/cell) in several human tissues (colon, prostate, placenta, and uterus) and its expression pattern seems to be conserved from development to adulthood (Sasaki et al., 2007; Wang et al., 2011). A recent study based on 156 CRC and 21 adjacent non-malignant tissues showed that *HOTTIP* is expressed at significantly higher levels in CRC than normal tissues (Ren et al., 2015). Interestingly, its expression is correlated positively and significantly with T stage, clinical stage and distant metastasis. Finally, the same authors indicate that the increased expression of *HOTTIP* may be used as an unfavorable prognosis predictor for CRC patients. A more recent study confirmed that *HOTTIP* is involved in CRC (Lian et al., 2015). The authors observed that the over-expression of *HOTTIP* is correlated with advanced pathological stage and larger tumor size. Interestingly, the knockdown of *HOTTIP in vivo* inhibited the growth of the tumor, suggesting that *HOTTIP* may be a good candidate for therapy of CRC.

### LncRNA-ATB

A recent exploratory analysis performed in hepatocellular carcinoma cell lines identified a new lncRNA altered upon *TGF*-β activation, named lncRNA-activated by *TGF*-β (*lncRNA-ATB*), that promotes invasion and metastasis through epithelial-mesenchymal transition (Yuan et al., 2014). Characterization analyses found that *lncRNA-ATB* is 2446 bp long and a BLAST search of the sequence against the human genome showed the presence of three highly homologous regions mapping on chromosomes 13, 14, and 22. To further elucidate the functional role of *lncRNA-ATB* in CRC, and its implication in invasion and metastasis, a retrospective study used RT-qPCR to determine the expression levels of *lncRNA-ATB* in a CRC tissue dataset. The authors found that *lncRNA-ATB* expression was significantly associated with tumor size, depth, lymphatic invasion, vascular invasion, and lymph node metastasis. Therefore, *lncRNA-ATB* may be involved in the progression of CRC and may be a prospective biomarker for invasion and metastasis in CRC. Although this result needs further validation in a larger cohort, *lncRNA-ATB* could represent a molecular target for controlling invasion-metastasis cascade in CRC (Iguchi et al., 2015).

### MALAT1

Metastasis Associated Lung Adenocarcinoma Transcript 1 (*MALAT1*) is one of the most promising lncRNA in CRC prognosis. It is an 8.75 kb long non-coding RNA transcribed from the nuclear enriched transcript-2 (*NEAT-2)* gene, located in chromosome 11q13. Initially associated to non-small cell lung cancer metastasis, the over-expression of *MALAT1* has been further described in metastasis of various tumors as hepatocellular carcinoma and endometrial stromal sarcoma (Ji et al., 2003; Smolle et al., 2014). Initial *in vitro* studies performed in CRC cell lines reported alterations in two fragments of the lncRNA transcript. First, the over-expression of fragment 6918–8441 nt that leads to proliferation and invasion promotion in CRC cells lines. Second, a deletion of 5434–6951 nt fragment that includes a key factor for normal cell functioning, and whose absence contributes to carcinogenic processes (Xu et al., 2011). Next, Yang et al. (2015) confirmed that *MALAT1* expression was higher in human CRC cell lines derived from metastasis compared to cells derived from primary CRC tissue. Moreover, over-expression of the activated transcript in SW480 cells promoted cell colony formation, migration and invasion mediated by PRKA (kinase anchor protein 9). The authors also reported that *MALAT1* expression was significantly higher in tumors than in their matching normal tissues especially in CRC tissues with lymph node metastasis (Yang et al., 2015). In this regard, another study carried out using RT-qPCR and human CRC tissues (stage II/III) confirmed that *MALAT1* expression was 2.26 times higher (*P* = 0.0004) in tumor vs. non-cancerous tissue (Zheng et al., 2014). Besides, association analyses of *MALAT1* expression and patient's prognostic factors such as disease free survival and overall survival indicated that *MALAT1* over-expression is correlated with poor prognosis in CRC patients, whereas multi-variate analysis confirmed *MALAT1* as an independent prognostic risk factor. Recently, a meta-analysis including 1216 participants reported a correlation between high *MALAT1* expression and three markers of cancer progression: lymph node metastasis, distant metastasis and survival rate (Wu et al., 2015). Overall, these data point to *MALAT1* as a potential independent marker of cancer metastasis and prognosis and as a potential molecular target to control cancer metastasis.

### PCAT-1

Prostate cancer-associated ncRNA transcript 1 (*PCAT-1)* is a long intergenic non-coding RNA codified by a gene located in a gene desert of chromosome 8q24, about 725 kb from the *c-MYC* oncogene. *PCAT-1* was discovered in 2011 by RNA-sequencing and is implicated in prostate cancer progression (Prensner et al., 2011). *PCAT-1* is up-regulated in prostate cancer tumor tissues and it has been shown to promote cell proliferation through association with polycomb repressive complex 2 (*PCR2*) as a transcriptional repressor. Moreover, down-regulation of *PCAT-1* by interference RNA was enough to reduce cell proliferation *in vitro*, pointing to *PCAT-1* as a potential therapeutic target of prostate cancer (Prensner et al., 2014). So far, *PCAT-1* expression has been associated to few cancer types such as CRC, and esophageal squamous carcinoma (Shi et al., 2015). Ge et al. (2013) explored the potential use of *PCAT-1* as a biomarker for CRC diagnosis and therapy. For this, they analyzed the expression levels of *PCAT-1* by Real Time qPCR in 108 CRC tumors and 81 matched controls (selected from 2003 to 2007), and observed that *PCAT-1* was frequently up-regulated in CRC tissues compared with normal adjacent tissue, and that *PCAT-1* was able to stratify overall survival and correlated with distant metastasis in CRC patients. In addition, the over-expression was unrelated to an increment of *PCAT-1* gene copy number variants. Multivariate analysis confirmed that *PCAT-1* was an independent prognostic factor (Ge et al., 2013). Therefore, *PCAT-1* may be considered as a novel prognostic biomarker. Since CRC prognosis may be influenced by *PCAT-1*, the authors suggested that, once elucidated, the molecular mechanisms by which *PCAT-1* is involved in CRC progression may be useful as an adjuvant therapy target.

### PVT1

The human *PVT1* oncogene (non-protein coding) is located at 8q24. A wide variety of solid tumors carry amplification of the 8q24 locus. Gain of supernumerary copies of the 8q24 chromosomal region has been shown to be common in many human cancers and is associated with poor prognosis. Over-expression of *PVT1* has been suggested as a powerful predictor of tumor progression and patient survival in a diverse range of cancer types, such as pancreatic cancer (Huang et al., 2015), gastric cancer (Kong et al., 2015), hepatocellular cancer (Ding et al., 2015), ovarian and breast cancer (Guan et al., 2007). Another remarkable feature of the *PVT1 l*ocus is that it resides about 60 kb apart from the 3-prime of the well-known *MYC* oncogene and that these two genes are co-amplified in CRC cell lines (Shtivelman and Bishop, 1989; Tseng et al., 2014).

Takahashi et al. (2014) showed that *PVT1* expression levels in colorectal cancer tissues were significantly higher than those in non-cancerous tissues. Knockdown of *PVT1* significantly inhibited colorectal cancer cells proliferation and reduced invasive abilities compared with negative control cells. Moreover, down-regulated *PVT1* promotes colorectal cancer cells apoptosis. The authors found that colorectal cancer patients with high *PVT1* expression had a significantly poorer prognosis than those with low P*VT1* expression (*P* = 0.0101). Univariate and multivariate analyses showed that *PVT1* expression level was an independent prognostic indicator of overall survival in patients with CRC (relative risk: 2.532 *P* = 0.016; Takahashi et al., 2014).

### TUG1

Taurine up-regulated gene 1 (*TUG1*) is a 9.7 kb gene mapping to chromosome 22q12 that codifies a long non-coding RNA. *TUG1* is highly conserved in mammals, and was originally detected in a genomic screen in taurine treated mouse retinal cells (Young et al., 2005). In this study, the authors found that *TUG1* knockdown resulted in malformed or non-existent outer segments of transfected photoreceptors through increased apoptosis in the newborn retina. In humans, elevated levels of *TUG1* have been found in the caudate nucleus of patients suffering from Huntington's disease (Johnson, 2012), whereas additional studies showed that lncRNA *TUG1* was involved in cancer progression, affecting apoptosis and proliferation in several human tumor cells. Zhang et al. (2013) proved that lncRNA *TUG1* is generally up-regulated in osteosarcoma and regulates proliferation and apoptosis of osteosarcoma cells. Han et al. (2013) reported that high *TUG1* expression levels were associated with high grade and stage of urothelial carcinoma of the bladder, whereas its silencing led to cell proliferation inhibition and apoptosis induction. Recently, another study (Sun et al., 2016) suggested that *TUG1* could play a critical role in CRC metastasis, and that it may serve both as a prognostic biomarker and therapeutic target. After analyzing the expression levels of *TUG1* in 120 CRC patients, the authors observed an up-regulation of the lncRNA in tumor tissue that in addition was closely correlated with the survival time of the CRC patients. Further *in vitro* analyses showed the oncogenic effect of *TUG1* over-expression in CRC cell lines. *TUG1* over-expression increased colony formation, migration and metastatic potential *in vivo*. Besides, the authors showed that *TUG1* activated EMT-related gene expression.

### UCA1

Urothelial Cancer Associated 1 (*UCA1*) is a lncRNA originally identified in bladder transitional cell carcinoma (Wang et al., 2006). The sequence of 7.37 kb is located in 19p13 chromosome. The proliferative, migratory, invasive, and drug resistance behaviors of human bladder TCC cell line BLS-211 were enhanced by exogenous *UCA1* expression *in vitro* (Wang et al., 2006). *UCA1* has been found significantly up-regulated in most tumors and cancer cells, including colorectal cancer. A multivariate survival analysis also indicated that *UCA1* could be an independent prognostic marker in gastric cancer. Han and collaborators found that *UCA1* levels were markedly increased in colorectal cancer tissues and cells, and that they influenced proliferation, apoptosis and cell cycle progression of colorectal cancer cells (Han et al., 2014). In addition, the authors assessed the correlation between *UCA1* expression and various clinicopathological data. They demonstrated that patients with high *UCA1* expression levels had a larger tumor size, less differentiated histology and greater tumor depth as compared to the low *UCA1* expression group. Furthermore, patients with high *UCA1* expression had a significantly poorer prognosis compared to patients with low *UCA1* expression (*P* = 0.046). Recently, Ni and colleagues reported that the expression of *UCA1* was statistically correlated with lymph node metastasis, distant metastasis and tumor stage. Finally, patients with high *UCA1* expression had a poor prognosis and multivariate analysis indicated that *UCA1* over-expression is an independent predictor for CRC (Ni et al., 2015).

## Tumor suppressive LncRNAs associated with prognosis in colorectal cancer

### GAS5

First described as a tumor suppressor in 1988, the Growth Arrest-Specific transcript 5 (*GAS5*) is a 630 nt long non-coding transcript transcribed by a gene located in chromosome 1q25 (Schneider et al., 1988). Previous studies showed that *GAS5* was able to bind the glucocorticoid receptor (GR) in growth arrested cells, influencing cell survival, metabolic regulation (Kino et al., 2010), and apoptosis (Mourtada-Maarabouni et al., 2008) when over-expressed. Conversely, *GAS5* down-regulation has been related to different types of cancer such as breast (Mourtada-Maarabouni et al., 2009), prostate (Pickard et al., 2013), renal cancer (Qiao et al., 2013), and more recently to cervical (Cao et al., 2014), and gastric cancer (Guo X. et al., 2015). Regarding CRC, Yin et al. (2014) observed that *GAS5* is down-regulated in human CRC tissues compared to the adjacent normal lung tissues, and that lower levels of *GAS5* are correlated with larger tumor size, low histological grade and advanced TNM stage, consistent with previous observations. Furthermore, the analysis of *GAS5* expression and prognosis showed that patients with low levels of *GAS5* had remarkably shorter survival time than those with high levels (*P* < 0.001). Multivariate analysis revealed that *GAS5* expression was a significant independent predictor of poor survival of CRC patients (*P* = 0.034), suggesting an important role of *GAS5* in CRC development and progression. In order to confirm these results, the authors tested the effect of *GAS5* over-expression both *in vitro* (HCT-116 and DLD-1) and *in vivo* (CRC xenografts mice) and observed that over-expression of *GAS5* could inhibit cell proliferation (Yin et al., 2014).

### LINC01296

*LINC01296* was identified by Qiu and Yan (2015) after re**-analyzing five Affymetrix Human Exon 1.0 ST published datasets from Gene Expression Omnibus (GEO). In total 355 samples were chosen to identify differentially expressed lncRNAs and their potential as diagnostic biomarkers for CRC. Twenty-five lncRNAs were differentially expressed between CRC tissue and tumor-adjacent normal tissue samples. Among them, *LINC01296* was shown to be significantly associated with the overall survival of patients with CRC. Univariate and multivariate analysis between expression levels of *LINC01296* and TNM stages as well as MSI (microsatellite instability) status indicated that *LINC01296* was a significant predictor of survival in CRC (*P* = 0.001) in addition to TNM stage (*P* = 0.001) and MSI status (*P* = 0.006). These results were validated in an independent series suggesting that *LINC01296* could be a novel prognostic biomarker for CRC (Qiu and Yan, 2015).

### MEG3

Maternally Expressed Gene 3 (*MEG3*) gene is located in chromosome 14q32, encodes a lncRNA and belongs to the imprinted *DLK1-MEG3* locus which contains some maternally and paternally imprinted genes (Benetatos et al., 2011). It is expressed in many normal tissues but multiple mechanisms contribute to the loss of *MEG3* expression in tumors, including gene dosage and epigenetic changes. Strong evidence exists to support that *MEG3* is a lncRNA tumor suppressor (Zhou et al., 2012). Yin et al. (2015) found that *MEG3* expression is down-regulated in CRC tumor tissue and that may regulate CRC cell proliferation *in vitro* and *in vivo*. They also demonstrated that *MEG3* levels were remarkably correlated with histological grade, deeper tumor invasion, and advanced TNM stage (*P* < 0.001). Univariate and multivariate analyses show *MEG3* expression as an independent predictor for overall survival (Yin et al., 2015).

### ncRAN

Non-coding RNA expressed in Aggressive Neuroblastoma (*ncRAN)* is a long non-coding transcript mapping to chromosome 17q25, a region of frequent gains in neuroblastoma that harbors two transcripts (Nbla10727, Nbla12061). Initially, it has been reported that high levels of *ncRAN* lncRNA were significantly associated with poor neuroblastoma patient's outcome (Yu et al., 2009), as well as enhanced cell proliferation, migration and invasion in bladder cancer cell lines (Zhu et al., 2011). Conversely, recent studies performed in CRC reported down-regulation of *ncRAN* in CRC fresh tumors and cell lines compared with normal adjacent tissue and normal intestinal mucosa cell line. Besides, reduced expression of *ncRAN* was associated with poorly differentiated tumors, liver metastasis and reduced overall survival rate. *In vitro* studies showed that *ncRAN* may mediate cell invasion and migration of CRC cells with little or no effect in cell proliferation, thus pointing to *ncRAN* as a new potential early diagnostic biomarker (Smolle et al., 2014; Qi et al., 2015). Overall, there is strong evidence that the expression pattern of *ncRNA* is tumor-dependent.

### ncRuPAR

*ncRuPAR* is a newly discovered long noncoding RNA molecule, located in 5q13 chromosome, that can up-regulate protease-activated receptor-1 (*PAR-1*) during embryonic growth. *PAR-1* has been implicated in tumor growth, invasion, and metastasis in several malignancies including colonic adenocarcinoma (Dorsam and Gutkind, 2007; Adams et al., 2015). Up-regulation of *PAR-1* expression has been found in gastric cancer (Liu et al., 2014), invasive breast cancer, colon cancer (Darmoul et al., 2003), and advanced stage prostate cancer (Wang et al., 2014). Adams et al. (2015) established a functional role for thrombin and its targets *PAR-1* and fibrinogen in the pathogenesis of colonic adenocarcinoma, supporting tumor growth as well as local invasion and metastasis (Adams et al., 2015). Recently, the study of Yan et al. (2014) found that *ncRuPAR* expression in CRC tissues was statistically lower than in adjacent normal tissue. Moreover, the authors reported that expression of *ncRuPAR* was significantly correlated with lymph node metastasis, distant metastasis, Dukes' stage (Haq et al., 2009), differentiation, and TNM stage (Yan et al., 2014). These findings suggest that *ncRuPAR* could act as a biomarker for progression and prognosis of CRC.

### RP11-462C24.1

*RP11-462C24.1* is a long non-coding RNA consisting of four exons with a length of 1136 bp, located in chromosome 4q25 and with an unknown function. A prospective analysis screened the aberrant expression of lncRNAs comparing CRC tumor tissues and normal adjacent tissues, as well as CRC metastatic tumors and non-metastatic tumors (Shi et al., 2014). Six lncRNAs were selected for RT-qPCR validation in an independent cohort of 86 CRC patients, based on their genomic location and expression levels. Then, the authors explored the association of *RP11-462C24.1* expression with clinicopathological factors. The analysis showed a decreased expression of the lncRNA *RP11-462C24.1* in tumor tissues compared with normal adjacent tissue (*P* < 0.001). Moreover, *RP11-462C24.1* expression was also lower in CRC patients with metastasis than those without metastasis (*P* < 0.049), and its low expression significantly correlated with more distant metastasis (*P* < 0.011). Multivariate analysis identified *RP11-462C24.1* as an independent prognostic factor for CRC (*P* < 0.005), whereas Kaplan-Meier analysis showed that patients with low expression of *RP11-462C24.1* had a poor disease-free survival (*P* < 0.001). Overall, *RP11-462C24.1* could be considered a potential prognostic biomarker, providing a new strategy for diagnosis. The study also implicates *RP11-462C24.1* in the tumorigenesis and progression of CRC, and identifies it as a potential target for further therapeutic studies (Shi et al., 2014).

### TUSC7

*TUSC7*, also known as *LOC285194*, is a lncRNA of 2105 nt in length located in chr3q13.31. It was first reported in osteosarcoma samples and cell lines, where depletion of *TUSC7* promoted proliferation of normal osteoblasts by regulation of apoptotic genes and cell cycle transcripts. Recent studies showed a potential tumor-suppressor role of this lncRNA in several cancers such as esophageal squamous cell carcinoma (Tong et al., 2014), pancreatic ductal adenocarcinoma (Ding et al., 2014) and colorectal cancer (Qi et al., 2013). Liu et al. (2013) showed that *TUSC7* is a direct transcription target of p53, and that ectopic expression of *TUSC7* inhibits tumor cell growth both *in vitro* and *in vivo*. Moreover, the authors determined the expression of *TUSC7* in colon cancer tissue by microarrays and showed that *TUSC7* was down-regulated in tumor specimens compared with normal specimens. Finally, Qi et al. (2013) demonstrated that relative expression levels of *TUSC7* were lower in a cohort of 81 tumor tissues from CRC patients and CRC cell lines compared to adjacent normal tissues and normal intestinal mucous cell line. The authors also assessed the correlation between *TUSC7* expression and clinicopathological data in CRC patients, and found that low expression of *TUSC7* was correlated with larger tumor size, higher tumor stage, and more distant metastasis. In addition, Kaplan-Meier analysis showed that patients with low *TUSC7* expression had a significantly poorer prognosis than those with high *TUSC7* expression (*P* = 0.010). All these data indicate that this lncRNA could represent a good candidate to consider as a potential biomarker for CRC prognosis and development.

## Conclusions and future perspectives

Colorectal cancer is one of the most commonly diagnosed cancers worldwide, and approximately half of all CRC patients die from distant metastasis. Thus, the key to improve CRC patients' survival is early diagnosis and early prediction of future metastasis. Diagnosis and prognosis markers are required to select the best-suited treatment under preoperative or postoperative conditions.

Advances in lncRNA expression profiling in human cancer have highlighted their potential value as tumor biomarkers in patient diagnosis and prognosis. Previous studies showed that lncRNAs play an important role in regulating gene expression at various levels, including chromatin modification, transcription, and post-transcriptional processing. In addition, lncRNAs have been reported to have tumor suppressor and oncogenic roles. In particular, levels of several lncRNAs are correlated with TNM stage, lymph node involvement, distant metastasis and overall survival. This data indicates that lncRNAs could offer clinical applications, both as diagnostic and prognostic markers, but also as novel specific therapeutic targets.

Another advantage of lncRNAs is that they may be used in clinics as minimally-invasive biomarkers due to their presence in body fluids, such as urine and plasma or serum. For example, *HOTAIR* and *MALAT1* have been reported as diagnostic and prognostic markers in the serum/plasma of cancer patients (Svoboda et al., 2014; Kishikawa et al., 2015). In plasma, *H19* is differentially expressed in patients with gastric cancer compared to healthy controls (Arita et al., 2013). Future studies on circulating lncRNAs would allow us to identify lncRNAs as putative minimally-invasive biomarkers for CRC. In this regard, a challenging aspect is the low expression levels of some lncRNAs, which brings about the need for using protocols based on amplification and/or enrichment of the circulating molecules.

It is important to bear in mind that, although in this review we have only focused and comprehensively described the lncRNAs significantly associated to CRC prognosis, other lncRNAs previously found dysregulated in CRC patients or associated to prognosis in other types of cancer should also be considered in future studies when looking for biomarkers of CRC prognosis. Among them, for example, Colon Cancer Associated Transcript 2 (*CCAT2)* is overexpressed in microsatellite stable CRC samples (Ling et al., 2013). Although a recent study failed to detect *CCAT2* in human CRC tissue or CRC cell lines (Xiang et al., 2014), elevated expression of *CCAT2* has been associated with poor prognosis in a wide range of cancers, including breast cancer (Redis et al., 2013), non-small cell lung cancer (Qiu et al., 2014), gastric cancer (Wang C. Y. et al., 2015), and esophageal squamous cell carcinoma (Zhang et al., 2015). Another well-established up-regulated lncRNA associated with CRC and other cancer types is Colorectal Neoplasia Differentially Expressed gene (*CRNDE*). *CRNDE* is activated during early steps of CRC development, and its expression is highly elevated in colorectal adenomas and adenocarcinomas (Graham et al., 2011). *CRNDE* was also reported to promote glioma cell growth and invasion, being highly up-regulated both in patients with glioma and *in vitro* cell lines (Wang Y. et al., 2015), and a recent study showed evidence of *CRNDE* as a potential marker of poor prognosis in women with ovarian carcinomas, with different outcomes depending on the therapeutic regimen used (Szafron et al., 2015). In addition, a very recent study (Esposti et al., 2016) confirmed *CRNDE* as well as lncRNA *H19*, among other known and new oncogenic lncRNAs, to be up-regulated in patients with hepatocellular carcinoma when performing RNA-Seq analyses in 23 liver tissues (controls, cirrhotic and hepatocellular carcinomas). Indeed, increased levels of the *H19* transcript have been observed in many cancers, including CRC (Liang et al., 2015), suggesting that *H19* and its gene products may be involved in tumor progression although prognostic value has not been proven so far. Thus, apart from the reviewed lncRNAs clearly linked to CRC prognosis, several studies have identified a large number of other lncRNAs that are dysregulated in CRC (reviewed in Ragusa et al., 2015; Xie et al., 2015). Since they may be potential prognostic biomarkers once their sensitivities and specificities are established, we think they should be taken into account in future analyses studying the possible role and use of lncRNAs as prognostic biomarkers in CRC.

Taken together, the use of lncRNAs as CRC prognostic biomarkers seems very promising. In the future, further characterization of these lncRNAs and greater understanding of their molecular function may support their clinical use for improving cancer diagnosis, monitoring cancer progression and as targeted therapies.

## Author contributions

ES, ABV, and SIG authors make substantial contributions to conception, design, and acquisition of data. Participate in drafting the article, tables and the figure. CP, TG, and C. Pericay revised the article critically for important intellectual content and give final approval of the version to be submitted. ES, ABV and SIG authors have equal contributions. TG and C. Pericay share senior authorship and correspondence.

### Conflict of interest statement

The authors declare that the research was conducted in the absence of any commercial or financial relationships that could be construed as a potential conflict of interest.
